# How Academics Face the World: A Study of 5829 Homepage Pictures

**DOI:** 10.1371/journal.pone.0038940

**Published:** 2012-07-17

**Authors:** Owen Churches, Rebecca Callahan, Dana Michalski, Nicola Brewer, Emma Turner, Hannah Amy Diane Keage, Nicole Annette Thomas, Mike Elmo Richard Nicholls

**Affiliations:** 1 Cognitive Neuroscience Laboratory, School of Psychology, Social Work and Social Policy, University of South Australia, Adelaide, South Australia, Australia; 2 Hawke Research Institute, University of South Australia, Adelaide, South Australia, Australia; 3 Brain and Cognition Laboratory, School of Psychology, Flinders University, Adelaide, South Australia, Australia; George Mason University / Krasnow Institute for Advanced Study, United States of America

## Abstract

It is now standard practice, at Universities around the world, for academics to place pictures of themselves on a personal profile page maintained as part of their University’s web-site. Here we investigated what these pictures reveal about the way academics see themselves. Since there is an asymmetry in the degree to which emotional information is conveyed by the face, with the left side being more expressive than the right, we hypothesised that academics in the sciences would seek to pose as non-emotional rationalists and put their right cheek forward, while academics in the arts would express their emotionality and pose with the left cheek forward. We sourced 5829 pictures of academics from their University websites and found that, consistent with the hypotheses, there was a significant difference in the direction of face posing between science academics and English academics with English academics showing a more leftward orientation. Academics in the Fine Arts and Performing Arts however, did not show the expected left cheek forward bias. We also analysed profile pictures of psychology academics and found a greater bias toward presenting the left check compared to science academics which makes psychologists appear more like arts academics than scientists. These findings indicate that the personal website pictures of academics mirror the cultural perceptions of emotional expressiveness across disciplines.

## Introduction

In 2010, Lindell and Savill [1] reported that, if a viewer is asked to determine from a portrait whether the person pictured is a student of Chemistry, English or Psychology, their decision can be predicted from the side of the face shown in the portrait: pictures showing the right cheek are more likely to be classified by the viewer as Chemistry students, while pictures showing the left cheek are more likely to be classified as English students. No bias was found for portraits classified as being Psychology students. This bias in face posing was predicted from the finding that people posing with their right cheek facing the viewer are considered to be less emotionally expressive than people posing with their left cheek facing the viewer [2] and the literature showing that in the popular imagination, people studying a science, such as Chemistry, are considered to be less emotional than people studying an arts discipline, such as English [3].

That the two sides of the face are unequal in their emotional expressivity is an observation first attributed to Darwin, who noted in those around him a tendency to move the muscles on the left side of the face more than the right side of the face when expressing emotions [4]. This observational finding has since been confirmed by experimental [5] and physiological [6] results. This difference in the outward display of emotion across the two sides of the face suggests a difference in the inward role of the two cerebral hemispheres in the creation and analysis of the emotional display, since the facial muscles, innervated by cranial nerve VII, rely predominantly on connections with the contralateral motor cortex [7]. And indeed, patients with lesions that are limited to the right hemisphere show a decreased ability to recognise emotion compared to patients with damage that is limited to the left hemisphere [8].

Interestingly, this difference in neuro-anatomy creates a bias in the way people pose for photographs. When people are asked to pose for a photograph that will be used as a family portrait they tend to present their left cheek to the camera. Conversely, when people are asked to pose for a photograph that will be used as their official portrait as an eminent scientist, they tend to present their right cheek to the camera [9]. Furthermore, this experimental manipulation bears out in a study of real portraits. In an analysis of portraits painted over the last five hundred years, McManus and Humphrey [10] showed that most pictures present the subject showing the left cheek. This bias however, is absent in the catalogue of portraits of inductees into the Royal Society [9] and portraits of other scienctists [11].

**Figure 1:**
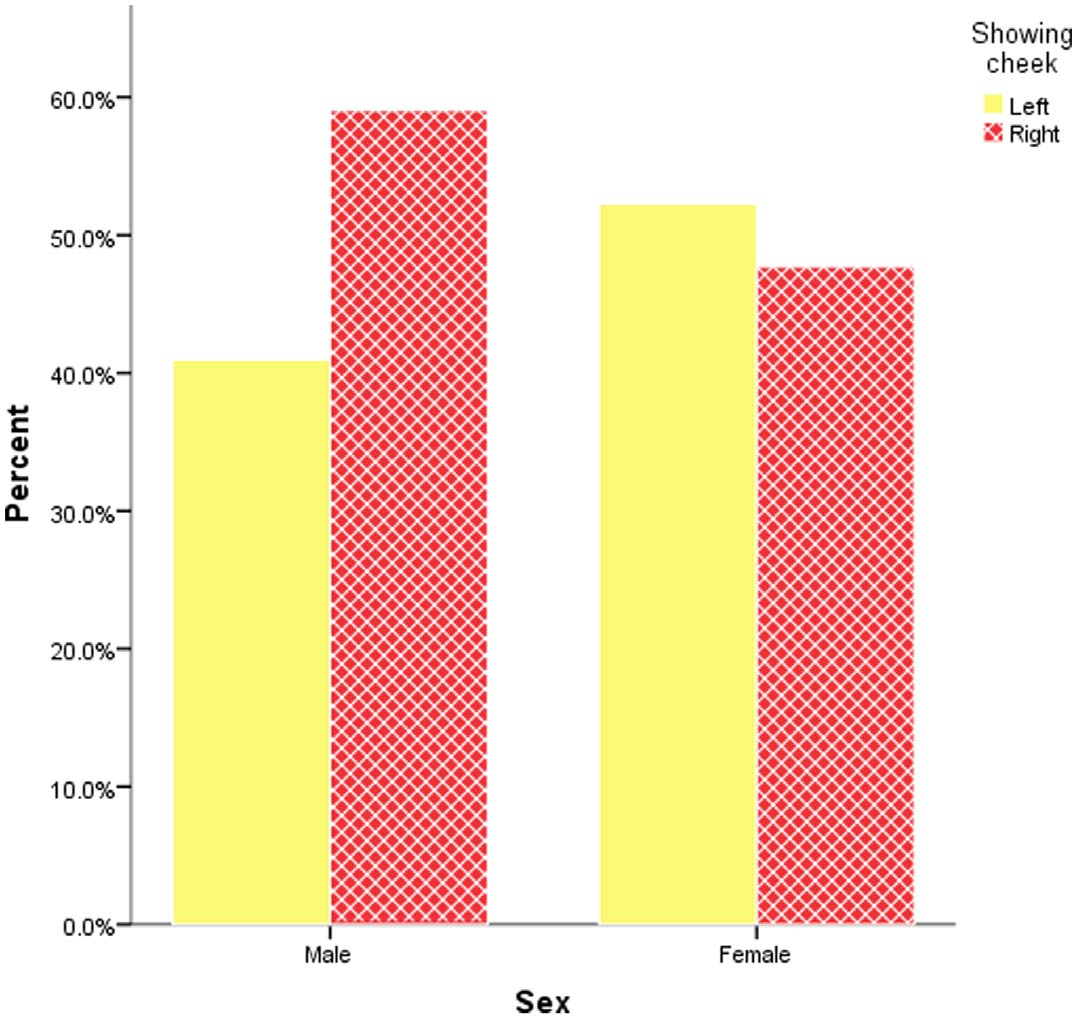
Face posing by sex.

So, if people posing for photographs showing their right cheek are more likely to be classified as science students and people posing for photographs showing their left cheek are more likely to be classified as arts students [1], how do professional academics in the arts and sciences choose to display themselves to the world via their most visible public picture: their personal homepage portrait housed on their University’s website? If scientists seek to appear objective and unemotional then they should be more likely to show the right cheek. Likewise, if arts academics seek to display their emotionality then they should be more likely to show their left cheek. But what of academic psychologists? Do psychologists present themselves primarily as the proponents of the scientific study of human behaviour and so pose with their right cheek forward? Or do they profess to be the willing receivers of problems and the promoters of a positive psyche posing with their left cheek forward? This study sought to investigate these questions via the systematic analysis of University webpages.

**Figure 2:**
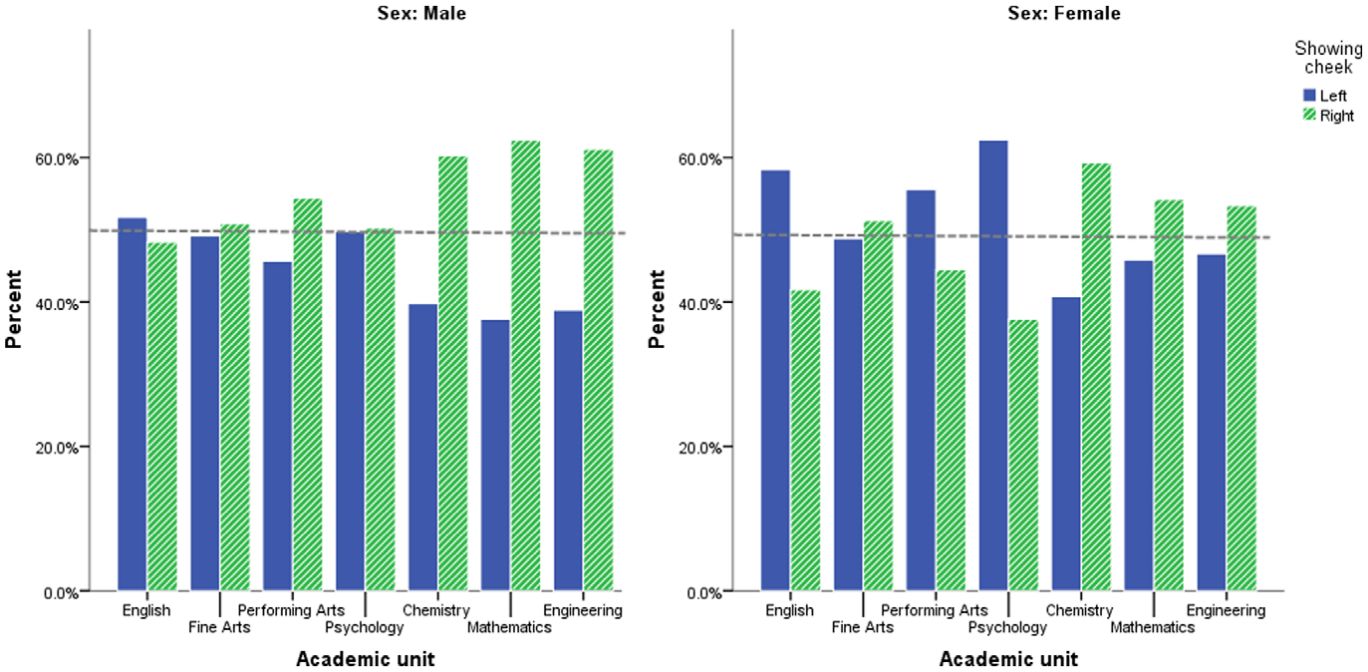
Face posing by academic unit and sex.

**Table 1:** Associations between academic units and face posing with sex controlled for.

Academic Unit	*n*	*B*(SE)	*Wald χ2*	*p*
English	292	−.51 (.13)	14.81	<.0001
Fine Arts	102	−.29 (.21)	1.97	.16
Performing Arts	84	−.31 (.23)	1.85	.17
Psychology	324	−.52 (.13)	16.98	<.0001
Chemistry	424	−.01 (.11)	<.000	.994
Mathematics	367	.049 (.12)	.17	.68
Engineering (referent)	1560			

## Results

Sex alone was a significant predictor of face posing angle, correctly predicting 58.5% of the cases (*χ2*(1) = 32.87, *p*<.0001) with males being more likely to show the right cheek and females being more likely to show the left cheek (see Figure 1). With sex controlled for, the model including academic unit predicted 59% of cases, including 21.73% of cases showing the left cheek and 87% of cases showing the right cheek (*χ2*(6) = 30.82, *p*<.0001). The results for each academic unit compared to Engineering are presented in Table 1 and Figure 2. These indicate that both English and Psychology academics showed a significantly different pattern of face posing (i.e. a more leftward direction) from Engineering academics with all other academic units showing non-significant differences from Engineering. After removing the 290 pictures thought to be professionally taken, the analyses showed the same pattern of results in which academic unit predicted 59.2% of cases, including 21.6% of cases showing the left cheek and 87.3% of cases showing the right cheek (*χ2*(6) = 25.77, *p*<.0001).

## Discussion

In this study we analysed the portraits of 5829 academics presented on their publicly accessible University profile page, to investigate whether scientists present themselves in their stereotyped role as objective rationalists and conceal their emotion by presenting the right cheek to the camera and conversely whether academics in the arts allow their emotions to be more visible by showing the left cheek. The results demonstrate that there is a clear difference in the way academics in the sciences and the arts present themselves to the world: scientists, including Engineers, Chemists and Mathematicians, tend to show the right cheek more than English academics. Thus scientists reduced the visibility of their emotions while English academics promoted the visibility of their emotions [2]. It is important to note that this effect was observed even when sex was controlled for statistically, since there is a large disparity between the proportion of male and female academics that make up arts and science faculties [12].

This difference between Chemistry and English academics is consistent with the findings of Lindell and Savill [1] who showed that models posing with their right cheek facing the camera are more likely to be thought of as Chemistry students, while models posing with their left cheek forward are more likely to be thought of as English students. Lindell and Savill also found that there was no bias in the way face posing predicted participants’ belief that the model was a Psychology student. However, our results show a bias for academic Psychologists to present the left cheek more than Engineering academics in their profile pictures. That is, academic psychologists readily display their emotion and thus appear more like arts academics than scientists.

This difference between our results and those of Lindell and Savill [1] may well reflect the difference between how academic Psychologists are seen by the public (in Lindell and Savill’s study) and how they see themselves (in our study). That is, it seems that the public perceive modern psychology as part way between an art and a science, reflecting the increasing role of neuroscience in the discipline [13], but that most academic psychologists, who may have entered the profession during its arts oriented past, perceive themselves as being more akin to arts academics than scientists. This effect is likely increased by the sample used by Lindell and Savill, which was not a full reflection of the general public but was limited to psychology students aged 18 to 24 years, a segment of the population particularly disposed to viewing psychology as a science, rather than an arts discipline [14]. This phenomenon could be studied further by investigating the subspecialisations of psychology and the year in which psychologists gained their PhD.

Interestingly, our results for academics in the Fine Arts and Performing Arts did not show the same bias toward presenting the left cheek shown by English and Psychology academics. This finding warrants further investigation. One explanation is that academics in the Fine Arts and Performing Arts are particularly familiar with the history and theory of portraiture, either as producers of portraits or as sitters. As such, it is possible that academics in the Fine Arts and Visual Arts are affected in the selection of their portrait picture by factors unconsidered by other academics. More research will be required to investigate this particular finding.

Our results also showed that sex alone was a significant predictor of face posing with male academics tending to show the right cheek and female academics tending to show the left cheek. However, Lindell and Savill [1] found no effect of the models’ sex when they asked participants to guess which discipline the different models where studying. Again, this may reflect a cultural change from the generation that are now students (in Lindell and Savill’s study) to the generation that are now academics (in our study). That is, current academics present themselves in stereotyped gender roles with males inhibiting the display of their emotion and females readily displaying their emotion but students aged 18 to 24 do not relate gender to emotional expressivity so readily. In support of this position, some other studies using an undergraduate student sample have found that males and females are equally susceptible to the increased emotional information provided by the left cheek in portraits [2] and are equally moved to present the left cheek when attempting to be maximally emotional [9], though one study has found that male and female undergraduates differ in the presentation of their cheeks when they are asked to pose as themselves [15].

That the sitter does not have complete control over their pose in a photograph is a perennial problem in research using sourced portraits [9]. Therefore, the potential role of the photographer in creating the effects observed in this study is hard to quantify. Some Universities allow staff to place any photograph of themselves on their personal profile page, while other Universities hire a professional photographer to photograph their staff. To address this issue, we identified portraits thought to be taken professionally and after removing them from the sample, re-analysed the data. The effects were consistent with the full sample analysis, indicating that these findings are not influenced by professional photography practices. This may be expected, since it is unlikely that portrait photographers would be blind to the discipline of the academic they were photographing. Hence, for professionally photographed portraits, it is possible that the photographer sought to present the academic in the pose appropriate to the cliché of rational scientist or emotional arts academic and colluded with their sitter, placing scientists with their right cheek forward and arts academics with their left cheek forward.

In this study we have shown that there are clear differences in the way academics in the sciences and arts present themselves in their publicly accessible University profile picture. Mathematicians, Chemists and Engineers tend to show the right cheek, thus reducing the observable emotionality, while English and Psychology academics show the left cheek, exacerbating the expression of emotion. Further research will be required to determine the consequences of this face posing preference. For instance: are student ratings of professors higher for academics who show their left cheek in their profile picture because they engender a feeling of approachability? Or, are academics who show the right cheek cited more because they are thought by other academics to display a more critical rationality? So, academics be warned: we present ourselves to our students and colleagues in our profile pictures and the way we do so may reveal more about ourselves than we think.

## Methods

### Procedure

Portraits of academics were sourced from official university web sites of the 200 Universities listed in the Times Higher Education World University Rankings for 2010–2011 [16]. A random sample of 30 Universities was taken from this list. The home page of each university academic unit (English, Fine Arts, Performing Arts, Psychology, Chemistry, Mathematics, and Engineering), was then found by entering the name of the University along with the name of the unit into ‘Google’. Links were then followed to locate a list of ‘faculty’ or ‘academic staff’ and each portrait available from this list was inspected.

Universities were excluded if the website for academic staff was not in English. Individual photographs were only included if the full face was clearly visible and if they were free of any additional people or objects (e.g. laboratory equipment or books) in the foreground. All drawn or computer generated pictures were excluded. In order to determine which cheek was shown by the posing angle of each academic, portraits were classified as having only the left, right or both sides of the nose visible. Two assessors rated each picture with a high inter-rater reliability (Cronbach’s alpha = .972). Pictures which the assessors rated differently were reviewed and a consensus was reached. The sex of the academic was also recorded.

Some University departments allow their staff to choose the picture that is posted on their home page while other departments use a professional photographer. Since the photographer may also influence the posing angle shown [9], the analyses were also run with the pictures that were thought to be professionally taken removed from the sample. Raters were blind to the academic unit that each picture was taken from.

### Analysis

Of the 5829 faces rated, 3168 were posing with either the left or right side of the nose visible and were used in the analyses. To investigate the relationship between academic unit and face posing angle, logistic regression was used with cheek showing (left, right) as the outcome variable and academic unit (English, Fine Arts, Drama, Psychology, Chemistry, Mathematics, Engineering) as the predictor variable. As Engineering had the largest sample size it was used as the referent to provide greater power to detect differences. Sex was used as a co-variate.
